# Characteristics in Stages of Change and Decisional Balance among Smokers: The Burden of Obstructive Lung Diseases (BOLD)-Australia Study

**DOI:** 10.3390/ijerph16183372

**Published:** 2019-09-12

**Authors:** Marsha A. Ivey, Graeme P. Maguire, Brett G. Toelle, Guy B. Marks, Michael J. Abramson, Richard Wood-Baker

**Affiliations:** 1 School of Public Health and Preventive Medicine, Monash University, Melbourne 3004, Australia; marsha.ivey@monash.edu; 2 Department of ParaClinical Sciences, Faculty of Medical Sciences, The University of the West Indies, St Augustine, Trinidad and Tobago; 3 Western Health, Footscray 3011, Australia; graeme.maguire@wh.org.au; 4 Respiratory and Environmental Epidemiology Group, Woolcock Institute of Medical Research, University of Sydney, Sydney 2037, Australia; brett.toelle@sydney.edu.au (B.G.T.); guy.marks@sydney.edu.au (G.B.M.); 5 Sydney Local Health District, Camperdown 2050, Australia; 6 South Western Sydney Clinical School, University of New South Wales, Sydney 2052, Australia; 7 School of Medicine, University of Tasmania, Hobart 7005, Australia; richard.woodbaker@utas.edu.au

**Keywords:** stages of change, decisional balance, smoking cessation, gender difference, Burden of Obstructive Lung Disease (BOLD)

## Abstract

Smoking cessation remains a health promotion target. Applying the Transtheoretical Model to Australian Burden of Obstructive Lung Diseases (BOLD) data, we examined differences in stages of change (SoC) and readiness to quit decisional behaviours. Factors were identified likely to influence readiness of smokers, ≥40 years old, to quit. Analysis was restricted to current smokers classified to one of three stages: pre-contemplation (PC), contemplation (C) or preparation (P) to quit. Their ability to balance positive and negative consequences was measured using decisional balance. Among 314 smokers, 43.0% females and 60.8% overweight/obese, the distribution of SoC was: 38.1% PC, 38.3% C and 23.5% P. Overweight/obesity was associated with readiness to quit in stages C and P and there were more negative than positive attitudes towards smoking in those stages. Males were significantly heavier smokers in PC and C stages. Females used smoking cessation medication more frequently in PC stage, were more embarrassed about smoking and had greater negative reinforcements from smoking. Age started smoking and factors related to smoking history were associated with readiness to quit and increased the odds of being in stage C or P. An overweight/obese smoker was likely to be contemplating or preparing to quit. In these stages, smokers have more negative attitudes toward smoking. Starting smoking later, taking advice on cessation from health providers and using quit medications indicate increased readiness to quit. Evaluating these factors in smokers and developing cessation gain-framed messages may prove useful to healthcare providers.

## 1. Introduction

Tobacco use is a global epidemic with related diseases estimated to kill over eight million people a year globally, which are mostly avoidable [1]. Unfortunately, the true effect on mortality is not immediate—usually taking years to manifest, making it a slow killer [2]. In Australia, smoking was reported to be the cause of 13.3% of deaths in 2015 and causally linked to the burden of 39 diseases [3].

Men and women are affected differently by tobacco use and tobacco messaging and smoking rates differ between men and women. There is evidence of reduced levels of pulmonary function in male smokers, higher smoking prevalence among males, while women have higher quit rates, lower quit maintenance and use more strategies to cope with smoking temptation [4,5], In Australia, smoking is the largest single preventable cause of death and disease accounting for 9.3% of the total burden of disease [3,6]. Australia’s Health 2018, reported daily smoking rates had decreased from 24.0% to 12.0% between 1991 and 2016 [6]. Smoking is a modifiable risk factor and prevention and cessation remains an important public health issue globally.

Cessation advice when tailored to an individuals’ readiness to quit, could be more effective than just providing broad, non-specific advice. For early cessation to be achieved, different strategies that appeal to behaviour change must be employed. According to Velicer et al., as much as 50% of premature mortality can be attributed to health behaviours and understanding those behaviours is critical to interventions [7]. The Transtheoretical Model (TTM) is widely used for examining health behaviour change in smokers by examining their stages of change (SoC) and decisional balance (DB) [8,9,10,11]. It is an integrative behaviour change model for intentional change focused on the decision making of individuals [7]. This concept the—“stages of change” forms a key element of the TTM, where a person cycles through six stages of change: pre-contemplation, contemplation, preparation, action, maintenance and termination [8,12].

Targeting cessation advice may be aided by understanding smokers’ SoC, their DB, that is, how the potential gains (pros) and losses (cons) are balanced to influence a decision [8,13]. Studies using the first three stages, pre-contemplation, contemplation and preparation, have reported most smokers are in the precontemplation and contemplation stages compared to those preparing to quit. The distribution for these varies with the US having 40%-40%-20%, Europe 70%-20%-10% and Australia 55%-34%-11% [8,9,10]. Prevalence among the stages is more heavily weighted to those not thinking about quitting and those likely to quit in the next six months. Using the concept of stages, different programmes have been implemented to target behaviour change, primarily targeting intervention such as, for example, personalised smoking cessation for callers to the Australian Quitline, stage-based self-help programmes or individual counselling [14,15]. These types of interventions have mixed results of being neither more nor less successful than standard interventions. However, Borland et al. highlight that in using the TTM, tailored schedules for feedback can be created to provide intensive support to the smoker and can assist movement through the stages to stable cessation state [15].

The TTM is a popular model and one of the many theories and theoretical constructs that has been applied to smoking cessation and other problem behaviours [3]. However, it is not without its critics. The arguments against, claim that the definitions used for the stages are arbitrary, categories are not qualitatively distinct and there was no peer-review of the development of the model [16,17]. As with most models, there are limitations and the TTM was not designed to be definitive or prescriptive. However, as a public health tool, TTM can be used to generate possible intervention strategies using stage-based activity promotion to address behaviour change at various stages of the decision-making process. Smoking, as with other health behaviours is complex and there are no “gold standard” algorithms for staging [18].

Despite decreasing smoking rates, Australia continues to make smoking cessation a key public health focus. TTM has come under criticism in the past decade, with for example Borland et al. “tweaking” the TTM to reconceive the stages of precontemplation and contemplation by considering important perspectives on change [19]. Nonetheless, these constructs still provide meaning full information for process change in smoking cessation. Our study was unable to capture the reconceived stages outlined by Borland et al. and thus examined factors likely to influence readiness to quit, readiness to quit decisional behaviours, change in SoC distribution and presence of differing demographics among Australian men and women who currently smoke.

## 2. Materials and Methods

### 2.1. Study Population

The study methodology for the Australian Burden of Obstructive Lung Disease (BOLD) study has been outlined elsewhere [20,21]. Briefly, the study comprised a representative sample of non-institutionalized Australians aged ≥40 years, residing in six locations across the country. Electoral rolls were used to obtain a sex-stratified, simple random sample of participants in all but one site. Sites were not randomly selected but chosen to reflect the country’s sociodemographic and geographic diversity [21]. A total of 3357 participated in the study but this analysis will focus on a subset of 314 participants who reported current cigarette smoking.

The BOLD-Australia Study was approved by the Human Research Ethics Committee of the University of Sydney (ref. no. 12-2006/9724). All sites obtained local ethics approval and participants provided written informed consent.

### 2.2. Measures

#### 2.2.1. Demographics and Smoking Characteristics

The BOLD study questionnaire collected information such as gender, age, education, weight and height, body mass index (BMI); smoking history: status, number of cigarettes per day, ages of starting and stopping, quit attempts, quit advice and quit medication; respiratory symptoms: cough, phlegm, wheeze and shortness of breath. Smoking status was established when participants were asked “Have you ever smoked cigarettes?” A “Yes” response meant “more than 20 packs of cigarettes in a lifetime or more than 1 cigarette each day for a year.” Ever smokers were further classified into current and former smokers by the age they stopped smoking.

#### 2.2.2. Stages of Change

The BOLD study used a specific questionnaire for stages of change among current smokers. The questionnaire only focused on three TTM stages. The revised algorithm for three of the five stages was used: pre-contemplation, contemplation and preparation [8,22]. Responses to “Are you seriously thinking of quitting smoking?” were used to group participants into the three stages as follows:

- Pre-contemplation: “No, not thinking of quitting”
  (individual is not ready/not thinking about making a change)
- Contemplation: “Yes, within the next 6 months”
  (individual thinking about making a change but not in the immediate future)
- Preparation: “Yes, within the next 30 days”
  (individual ready to change and intends to try to make a change in the immediate future and may be making small preparatory changes)

#### 2.2.3. Decisional Balance

The pros and cons for the DB were measured using a modified short form of the Smoking Decisional Balance Scale questionnaire [13]. This included responses to:

- Pros: “Smoking cigarettes relieves tension; Smoking helps me concentrate and do better work; I am relaxed and therefore more pleasant when smoking”
- Cons: “I’m embarrassed to have to smoke; My cigarette smoking bothers other people; People think I am foolish for ignoring the warnings about cigarette smoking”

A pro score and con score was calculated based on the mean of the 5-point Likert response scale (“1 = Not important” to “5 = Extremely important”). The DB was calculated as the difference between these mean pros and cons scores. Therefore, more positive or negative scores indicated positive or negative attitudes among smokers on the pro/con balance scale.

### 2.3. Statistical Analysis

Descriptive statistics were used to describe demographic and smoking characteristics. Differences between the SoC and gender differences within the stages were assessed using chi-square, *t*-test, Kruskal-Wallis and analysis of variance (ANOVA). Tukey’s adjustments were made for multiple comparisons. Multinomial logistic regression was used to determine factors that predicted smokers’ readiness to quit.

Analysis for the DB was affected by missing values. One study site, with 59 participants, was excluded as no data were collected. The demographic and smoking history profiles for this excluded group were not significantly different from other smokers, except for age where the excluded group was younger (51 vs. 54 years; *p =* 0.02). For this variable there were an additional 19 cases missing completely at random. Imputation using Expectation-Maximization was conducted and analysis performed on 255 cases [23].

For the logistic regression, the sub-sample of 255 cases with complete DB data was used. Purposeful selection was used for model building [24]. Models were fitted with one covariate at a time and assessed for fit. Checks were done for multicollinearity, correct ratio of cases to variables and outliers. Seven outlying cases were filtered out after examination of differences in classification accuracy. A multinomial logistic model was fitted to determine the odds of membership in a SoC. Pre-contemplation was used as the reference category when observing/predicting membership among the SoC. Two models were developed to compare individuals in the contemplation stage and preparation stage with those in the pre-contemplation stage. Usefulness of the model was determined by improvement in accuracy over chance alone. Smokers were in the contemplation or preparation stage if the OR > 1.0 and in the pre-contemplation stage if the OR < 1.0.

Level of statistical significance was set at 0.05, all tests were two-sided and SPSS (ver20, IBM, Armonk, NY, USA) was used to conduct the analysis.

## 3. Results

### 3.1. Demographics, Health Characteristics and Stages of Change

Of the 314 smokers, 43.0% were females, 94.6% Caucasian and 85.0% of working age. Participants ranged in age from 40-89 years (mean = 53, SD = 10) and the majority (60.8%) had a BMI > 25 kg/m^2^. Approximately 69.9% had 7–12 years of schooling and 26.6% over 12 years.

Respiratory symptoms were common: 45.2% usually had a cough without a cold; 34.7% usually brought up phlegm in the absence of a cold; 51.3% suffered from wheeze within the last 12 months; and 30.6% had dyspnoea on moderate exertion.

Smoking started at a mean age of 18 (SD = 5) years, with participants smoking a median of 15 (IQR: 10–20) cigarettes per day. Quit attempts of at least 24 hours had been made on average once (IQR: 0–3) in the previous 12 months. The majority (73.2%) indicated that they had previously received quit advice from a physician or other health care provider, about a quarter (25.5%) of whom had received the advice within the last 12 months. Nearly two fifths (36.9%) had used some type of medication in their previous quit attempts.

Approximately 38.1% of smokers were in pre-contemplation, 38.3% in contemplation and 23.5% in preparation stages (Table 1). The distributions of SoC among males and females showed no significant differences. Demographic, respiratory and smoking characteristics were examined across the SoC and within each stage by gender (Table 1). There were no significant gender differences within the SoC, nor overall significant associations observed between the SoC and the other demographic and health indicators except for BMI. BMI showed a significant difference between stages (*F*_2,305_ = 3.68, *p=* 0.03) with those overweight being more in the contemplation and preparation stages compared to pre-contemplation (*p =* 0.03 and *p* = 0.14 respectively).

Some associations are reported in Table 1 for when participants’ smoking history by SoC and gender were examined. The mean difference observed in starting age of smoking across the stages (*F*_2,307_ = 7.56, *p* = 0.001) with those in the preparation stage starting at an older age than those in contemplation and pre-contemplation (*p* = 0.04 and *p* < 0.001 respectively). However, within each stage, there was no significant difference between males and females for the age at which they started smoking. Based on the number of cigarettes smoked per day, males were heavier smokers than females in the pre-contemplation and contemplation stages (*U =* 1269, *p* = 0.02 and *U =* 1330, *p* = 0.04). The amount smoked in pack-years also appeared greater in men but this was only statistically significant in the pre-contemplation stage (*U* = 1231, *p* = 0.01). The median number of cigarettes smoked differed across the three stages (*χ*^2^_2,310_ = 9.4, *p* = 0.01); those in the preparation stage smoked less than those in contemplation (*p* = 0.002) and pre-contemplation (*p* = 0.03).

No gender or SoC differences were observed in smokers who received quit advice from a health professional. However, across the SoC, more participants in contemplation and preparation stages had received advice within the previous 12 months compared to those in pre-contemplation (*χ*^2^_2,227_ = 13.3, *p* = 0.001). There was a linear trend for participants to receive advice in later stages (*p* < 0.001). Additionally, of those who ever received quit advice, more females than males in the pre-contemplation stage used some form of medication to stop smoking (*χ*^2^_1, 84_ = 4.5, *p* = 0.03). While no significant difference in use of medication observed across the stages (*χ*^2^_2,227_ = 5.7, *p* = 0.06), there was a significant linear trend (*p* = 0.02). For quit attempts lasting a minimum of 24 hours in the previous year, there was a significant difference between individuals’ SoC (*χ*^2^_2,309_ = 5.7, *p* < 0.001), with most occurring in the preparation compared to contemplation stage (*p* < 0.001) and in the contemplation compared to the pre-contemplation stage (*p* = 0.01); no gender differences were observed within the SOC.

### 3.2. Decisional Balance and Stages of Change

In Table 2, there were no differences between males and females for the mean DB scores or the pro and con scores were observed. Examining individual responses related to the pro and con items, females had significantly higher scores when reporting smoking relieved tension (*t*_253_ = 2.7, *p* = 0.01) and that they were embarrassed to have to smoke (*t*_253_ = 2.2, *p* = 0.03).

On examining the relationship between DB and SoC, there were no gender differences observed for mean DB pro and con scores within each SoC stage. However, males displayed significant differences across the SoC for the DB cons score (*F*_2,138_ = 11.3, *p* < 0.001) and DB balance score (*F*_2,138_ = 7.4, *p* = 0.001). This was most noticeable amongst those in the contemplation and preparation stage, having greater aggregate scores when compared to those in pre-contemplation (*p*s < 0.0001) for the DB cons and similarly for the DB balance (*p =* 0.004 & *p =* 0.003 respectively). Females only displayed a significant difference across the stages for the cons (*F*_2,108_ = 6.9, *p* = 0.002); particularly between pre-contemplation and contemplation (*p* = 0.003) and pre-contemplation and preparation (*p* = 0.014). Additionally, there were more negative than positive attitudes towards smoking, primarily in the contemplation and preparation stages. Only the cons showed a significant difference in mean scores between the stages (*F*_2,249_ = 17.3, *p* < 0.001), with those in the contemplation and preparation stages reporting significantly more negative attitudes towards smoking than those in pre-contemplation (*p*s < 0.001). The balance of the pros and the cons showed an increasing trend in negative over positive attitudes toward smoking across the SoC (*F*_2,249_ = 9.2, *p* < 0.001).

### 3.3. Factors Associated with Readiness to Quit in Smokers

Table 3 presents the main independent effects of the variables entered into multivariable logistic regression models with the ORs showing the increased odds of a smoker being in a particular stage of change.

Quitting smoking for at least 24 h in the last 12 months was not a significant predictor. Those who had ever used some form of quit medication were more likely to be in the preparation stage compared to pre-contemplation and those provided with quit advice in the last 12 months were more likely to be in the contemplation and preparation stages than pre-contemplation. When the starting age increased by one year, smokers were 20.0% more likely to be in the contemplation stage and 39.0% more likely to be in the preparation stage than the pre-contemplation stage. DB showed that for every unit decrease in the con score, smokers were 35.0% more likely to be in contemplation and 32.0% in preparation than the pre-contemplation stage.

## 4. Discussion

In this study, we found being older (19 or 20 years) when starting smoking, using prescribed or over the counter cessation medications, receiving advice on cessation within a 12-month period and having more negative attitudes toward smoking were predictors of smokers contemplating or preparing to quit. Smokers preparing to quit tended to decrease their daily cigarette intake, increase their attempts at quitting (in the previous 12 months), received a higher frequency of cessation advice and used more cessation medications. Overweight and obese smokers were typically contemplating or preparing to quit. Gender differences observed were related to smoking history but were generally not prominent.

### 4.1. Predicting a Smokers Readiness to Quit

Smoking remains a major risk factor in Australia despite a decrease in smoking prevalence. In particular, the decline plateaued between 2013 and 2016 [6]; so it is important to continue work on reducing prevalence. We aimed to identify predictors of readiness to change in Australian smokers. These identified predictors could likely be utilised in technology-based health-related cessation tools as interventions that delay the start of early smoking among adolescents to improve likelihood of quitting at a later stage with the aid of medication and appropriate messaging. Health promotion and counselling programmes must remain dynamic, to effect behaviour change and integrate new findings related to stages of change may contribute to cessation efforts. The advent of online or e-tools with readily available health-related information changes the doctor-patient and healthcare-patient relationship; empowering patients to be more involved in the decision process [25]. Pre-determining a smoker’s readiness may be useful in building tailored cessation messages or programmes. This should also be incorporated when developing or updating health promotion messages using health-gain-framed statements. Interventions that at least delay the onset of smoking by adolescents may increase their likelihood of later quitting.

### 4.2. Stages of Change and Decisional Balance

Decisional balance weighs positive and negative aspects related to problem behaviours and then determines the importance of carrying out an action. Our study was fairly consistent with other studies, where the balance between the pros and cons of behaviour towards smoking varied according to the stage that a smoker occupied with change typically occurring in the contemplation and preparation stages [26,27,28,29]. Smokers did not seem to place much importance on the positives or negatives of smoking during the pre-contemplation stage but moving toward preparation there was greater endorsement of the negative effects associated with smoking behaviour. Essentially, the negatives of smoking were associated with smokers being likely to contemplate or prepare to quit; consistent with previous studies [26,27,28].

The importance of reinforcing the negative effects of smoking as a potential mechanism for driving smokers towards preparation provides support for current population health initiatives. The healthcare providers’ role is that of supporter and reinforcer but every opportunity should be taken to give cessation counsel, advice and follow-up whenever a smoker visits a health facility as few smokers visit specifically for a smoking complaint.

### 4.3. Stages of Change and Demographic and Health Characteristics

Findings were largely consistent with previous studies, with our stage distributions of the SoC being close to the theoretical expectation of 40-40-20 for PC-C-P, demonstrating most smokers are in the early stages [11,26,30]. This expectation was based primarily on US studies of population samples over 18 years of age and remains similar for those 40–64 years. Like the BOLD-US and other studies [5,31], gender did not play a role in stage distribution nor with the other key demographics and respiratory symptoms. European studies generally described a different distribution, 70-20-10, leaning more to the pre-contemplation stage [9]. However our stage distribution was different from other BOLD studies, which used similar methodology and age range and different from other Australian studies [11,14,30,32].

Borland and Balmford [19], applying a perspective-based TTM to Australian smokers, aged 18 to 40 years, found a 48-38-19 distribution while a randomised control trial reported a 11-54-45 distribution among smokers calling into the Victorian Quitline counselling service. Campbell et al. [32], found a stage distribution of 40-43-14 in indigenous Australians 15 years and older. Dissimilarities are likely related to population characteristics, smoking prevalence, policies, health promotion programmes and/or real differences in smokers’ readiness to quit. This part of the Australian BOLD study focused on non-indigenous smokers over 40 years old. Despite uncertainties around the use of the TTM, Borland and Balmford [19] and others have shown that the TTM is still applicable.

In Australia there has been a steady decline in smoking prevalence since 1995, which apart from smoking becoming increasingly unsociable, is associated with numerous mass media campaigns, increased taxes and plain packaging laws to discourage smoking and increase quitting [3]. Both Sweden and Iceland have also introduced laws and bans (between 2005 and 2007) on smoking in public places and restrictions on advertising and packaging [11,33]. However Kentucky (USA) does not have stringent anti-smoking campaigns, policies or enforce laws on advertising bans and taxes [34].

Similar to BOLD-Scandinavia, we found an association between body mass index and SoC [11], showing that overweight and obese smokers were contemplating or preparing to quit. Overweight and obesity is a global problem which, over the last two decades, has increased among Australian adults from 57% in 1995 to 63% in 2015 (36% overweight and 28% obese) [6]. Studies have found an increased risk of obesity among smokers, in addition to which as leading causes of chronic diseases, the combined effects could lead to worsening of conditions [35,36]. Changes in pulmonary function are also related to central obesity [37]. For healthcare providers, knowledge of presence of obesity or overweight among smokers is an additional indicator of likely lung function problems from the combined effects of these conditions [37]. Losing weight may be a good public health messages to incentivise improved lung function through quitting. However, there is a conundrum of smoking cessation also being associated with weight gain. Health providers and health promotion should be more aggressive in the presence of these combined risk factors and provide combined weight loss and tobacco messaging and treatments.

### 4.4. Stages of Change by Smoking History

The study indicates that the smoking history factors associated with readiness to quit were consistent with other studies, including BOLD [11,22,26,30,31,38].

Some smoking features also differed by gender with male pre-contemplators and contemplators consuming more cigarettes and more females using some form of quit medication when they were pre-contemplating. Young and Ward highlighted that Australian physicians were more likely to discuss smoking with male patients [39]. It was unclear if this resulted from men, because they smoke more, being more highly addicted and therefore should be encouraged to use quit aids like pharmacotherapy. Despite smoking being more predominant among males, with the morbidity of conditions related to tobacco smoking, clinicians should emphasize cessation to both genders. The Royal Australian College of General Practitioners(GP) guidelines note the important contribution that health professionals can make in cessation programmes by simply offering advice and assistance with quitting [40]. With over 80% of visits to a GP (or family/primary care physician), in a given year, their role in primary care smoking prevention is very important [41]. GPs identify approximately two thirds of smokers but only provide cessation advice to approximately half [42].

### 4.5. Strengths and Limitations

The main strength underlying these results is that the data are from a nationwide population sample [21]. The large sample of the main study allowed for reasonable estimates based on objective measures and the use of standardised definitions. The protocol and questionnaires were harmonised with the BOLD international protocol allowing for country comparisons [43].

However, this study is not without limitations. For example, the exclusion of one study site due to missing data may have an impact on the decisional balance analysis, despite their similar profile. Results should only be generalised to those aged ≥40 years and some participants may have provided answers deemed socially desirable. There may be some self-reporting bias and misclassification on the participant’s actual readiness to quit. Additionally, this study is cross-sectional, preventing inferences related to changes over time in TTM constructs.

## 5. Conclusions

In this study of 314 current smokers from the BOLD-Australia Study, we found quit attempts were more common among those preparing to quit while smoking advice and cessation medication decreased. Smokers displayed a range of common symptoms such as cough, phlegm wheeze and dyspnoea. Gender did not play a major role impacting SoC or DB but males received more advice on quitting in the preparation stages, while females used more cessation medications when pre-contemplating. The factors identified are modifiable and could be used by healthcare providers for cessation programmes; evaluating overweight/obese and using it to incentivise cessation, nudging smokers along the path to cessation with continued reinforcement of the negative impact of smoking while highlighting health gains. Progression through stages of change is not linear so re-evaluation of smoking behaviours and readiness to quit is important. Future research is needed to look at the comorbid link between obesity and smoking, two major modifiable risk factors, on cessation.

## Figures and Tables

**Table 1:** Demographics, Respiratory and Smoking Characteristics by Stages of Change and Gender, Burden of Obstructive Lung Diseases (BOLD)-Australia Study.

Characteristics	Stages of Change											
	Pre-Contemplation (*n* = 118)				Contemplation (*n* = 119)				Preparation (*n* = 73)			
	M	F	*p*	All	M	F	*p*	All	M	F	*p*	All
Age (mean, SD)	54.9 (10.0)	53.1 (10.2)	0.35	54.1 (10.1)	52.4 (8.6)	55.1 (9.6)	0.12	53.5 (9.1)	53.9 (11.2)	52.0 (8.4)	0.42	53.0 (10.0)
BMI (mean, SD)	25.1 (4.2)	26.3 (5.1)	0.15	**25.6 (4.6)**	27.6 (4.4)	26.8 (5.2)	0.38	**27.3 (4.7)**	27.9 (5.7)	26.1 (5.2)	0.16	**27.0 (5.5) ^‡^**
Years of Schooling (mean, SD)	11.7 (3.0)	11.2 (2.7)	0.41	11.5 (2.8)	11.4 (3.0)	11.9 (2.9)	0.43	11.4 (2.9)	11.5 (2.9)	11.8 (3.1)	0.68	11.6 (3.0)
Respiratory Symptoms (n, %)												
Cough	35.0 (52.2)	25.0 (49.0)	0.73	60.0 (50.8)	32.0 (45.1)	18.0 (37.5)	0.41	50.0 (42.0)	16.0 (42.1)	13.0 (37.1)	0.66	29.0 (39.7)
Phlegm	28.0 (41.8)	15.0 (29.4)	0.17	43.0 (36.8)	29.0 (40.8)	13.0 (27.1)	0.12	42.0 (35.3)	14.0 (36.8)	8.0 (22.8)	0.19	22.0 (30.1)
Wheeze in last 12 months	31.0 (46.3)	23.0 (45.1)	0.90	54.0 (45.8)	43.0 (60.6)	26.0 (54.2)	0.49	69.0 (58.0)	19.0 (50.0)	18.0 (51.4)	0.90	37.0 (50.7)
Dyspnoea on exertion	15.0 (23.8)	17.0 (34.0)	0.23	32.0 (27.4)	21.0 (31.8)	22.0 (46.8)	0.11	43.0 (38.1)	9.0 (24.3)	11.0 (34.4)	0.36	32.0 (28.3)
Smoking History												
Age started (mean, SD)	16.4 (3.0)	17.6 (4.2)	0.06	**16.9 (3.6)**	18.1 (4.7)	17.7 (4.2)	0.63	**18.0 (4.5)**	20.3 (7.7)	19.3 (6.7)	0.55	**19.8 (7.2) ^‡^**
Cigarettes/day (median, IQR)	20.0 (10.0, 25.0)	12.0 (5.0, 20.0)	**0.02 ^†^**	16.7 (7.0, 25.0) *	20.0 (12, 25)	15.0 (10.0, 20.0)	**0.04 ^†^**	**20.0 (10.0, 25.0)**	13.5 (5.0, 20.0)	10.0 (7.0, 15.0)	0.55	**10.0 (6.0, 20.0)**
Pack-Years (median, IQR)	30.2 (18.0, 47.2)	21.4 (5.5, 38.9)	**0.01 ^†^**	26.8 (12.2, 45.5) *	30.4 (18.7,44.3)	24.3 (14.2, 41.1)	0.17	**28.0 (17.2, 41.6)**	20.0 (8.2, 46.3)	17.2 (10.1, 24.9)	0.37	**18.1 (8.7, 37.4) ^‡^**
Quit Activity (n, %)												
Physician quit advice	52.0 (78.8)	32.0 (64.0)	0.08	84.0 (72.4)	55.0 (20.3)	35.0 (74.5)	0.51	90.0 (77.6)	28.0 (73.7)	25.0 (71.4)	0.83	53.0 (72.6)
^₴^ Advice in last 12 mo	11.0 (21.2)	6.0 (18.8)	0.79	17.0 (20.2) *	22.0 (40.0)	13.0 (37.1)	0.79	**35.0 (38.9)**	15.0 (53.6)	11.0 (44.0)	0.49	**26.0 (49.1) ^‡^**
^₴,ꬸ^ Quit medication	17.0 (32.7)	18.0 (56.2)	**0.03 ^†^**	35.0 (41.7) *	29.0 (52.7)	18.0 (51.4)	0.90	**47.0 (52.2)**	18.0 (64.3)	15.0 (60.0)	0.75	**33.0 (62.3)**
Quit attempts ≥ 24 h in last 12 mo (median, IQR)	0 (0, 1.0)	0 (0, 2.0)	0.54	0 (0, 1.0) *	1.0 (0, 2.0)	0.5 (0, 2.7)	0.98	**1.0 (0, 2.0)**	2.0 (1.0, 10.0)	5.0 (0, 12.0)	0.55	**3.0 (1, 11)**

* Fisher’s Exact Test; all bolded numbers are significant, ^†^ significant gender difference, within Stages of Change (*p* < 0.05); ^‡^ significant difference between Stages of Change (*p* < 0.05); ^₴^ a subset of those that responded to receiving physician advice; ꬸ prescribed or not * significant trend across Stages of Change (*p* < 0.05); mo—months, IQR—inter quartile range, SD—standard deviation.

**Table 2:** Decisional Balance and Response Items by Gender, BOLD-Australia Study.

Decisional Balance	Male (*n* = 143)		Female (*n* = 112)		*p*
	Mean	SD	Mean	SD	
Pro Score ^†^	7.0	2.7	7.6	2.9	0.11
Concentration Improved ^‡^	1.8	1.1	1.8	1.2	0.94
Relaxed and Pleasant	2.5	1.1	2.7	1.3	0.28
Tension Relief	2.7	1.2	3.1	1.2	**0.01**
Con Score	8.7	3.6	9.4	3.4	0.10
Bothers Others	3.4	1.4	3.5	1.3	0.51
Embarrassed	2.2	1.4	2.6	1.5	**0.03**
Foolish	3.1	1.6	3.3	1.5	0.24
Balance Score	−1.7	4.4	−1.9	3.7	0.72

^†^ Range for Scores: 3–15; ^‡^ Items based on a 5-point Likert response scale; significant values in bold.

**Table 3:** Smokers in Pre-contemplation Stage versus Contemplation and Preparation Stage of Smokers, BOLD-Australia Study.

Characteristics	Pre-Contemplationvs.			
	Contemplation		Preparation	
	OR	95% CI	OR	95% CI
Age started smoking	**1.20**	**1.05, 1.38**	**1.39**	**1.19, 1.62**
Con Score	**1.35**	**1.17, 1.55**	**1.32**	**1.12, 1.56**
Quit smoking at least 24 h in last 12 month	1.11	0.97, 1.26	1.14	0.99, 1.30
Quit medication used	2.21	0.94, 5.20	**6.15**	**1.99, 19.02**
Quit advice in last 12 month	**3.35**	**1.31, 8.56**	**4.45**	**1.47, 13.49**

*n* = 248 used for this analysis; OR – odds ratio; CI – confidence interval; significant values in bold.
